# Assessment of Serum Hypoxia Biomarkers Pre- and Post-radiotherapy in Patients with Brain Tumors

**DOI:** 10.1007/s12031-022-02065-z

**Published:** 2022-09-19

**Authors:** Sanaa A. El-Benhawy, Ola A. Sakr, Enayat I. Fahmy, Raed A. Ali, Mohamed S. Hussein, Esraa M. Nassar, Sherif M. Salem, Nehal Abu-Samra, Sherif Elzawawy

**Affiliations:** 1 Radiation Sciences Department, Medical Research Institute, Alexandria University, Alexandria, Egypt; 2 Cancer Management and Research Department, Medical Research Institute, Alexandria University, Alexandria, Egypt; 3 Radiology and Medical Imaging Department, Faculty of Technology of Medical Sciences, Baghdad University, Baghdad, Iraq; 4 Radiology Department, Faculty of Applied Medical Sciences, October 6 University, October, Egypt; 5 Department of Neurosurgery, Faculty of Medicine, Alexandria University, Alexandria, Egypt; 6 Department of Basic Sciences, Faculty of Physical Therapy, Pharos University, Alexandria, Egypt; 7 Clinical Oncology Department, Faculty of Medicine, Alexandria University, Alexandria, Egypt

**Keywords:** Hypoxia, HIF-1α, VEGF, Osteopontin, Erythropoietin, Meningioma, Glioblastoma

## Abstract

Hypoxia is a prevalent hallmark of many malignant neoplasms. The aim was to assess the serum hypoxia biomarkers HIF-1α, VEGF, osteopontin, erythropoietin, caveolin-1, GLUT-1, and LDH pre- and post-radiotherapy in patients with brain tumors. The study was conducted on 120 subjects were divided into two groups: group I: 40 healthy volunteers as control group. Group II: 80 brain tumor patients were subdivided into glioblastoma subgroup: 40 glioblastoma patients, meningioma subgroup: 40 malignant meningioma patients. Two venous blood samples were collected from every patient prior to and following RT and one sample from controls. Biomarkers were assayed by ELISA. In glioblastoma subgroup, HIF-1α, VEGF, and LDH were significantly increased after RT. On the contrary, these biomarkers were significantly decreased after RT in malignant meningioma subgroup. Osteopontin was significantly increased after RT in both subgroups. Regarding erythropoietin, it was significantly decreased in both subgroups when compared to before RT. Caveolin-1 showed a significant increase in glioblastoma subgroup after RT comparing to before RT. GLUT-1 was significantly increased after RT in both subgroups comparing to before RT. Association of significant elevation of hypoxia biomarkers either pre- or post-RT with aggressive tumor such as glioblastoma indicates that, they are markers of malignancy and may have a role in tumor development and progression.

## Introduction

Adult brain tumors are the second leading cause of cancer-related death (Khazaei et al. 2020). Because brain tumors are located in critical areas of central nervous system (CNS), traditional therapeutic interventions as example surgery, chemotherapy, and radiotherapy (RT) are difficult to use. As a result, innovative targeted alternative therapies are important for treating brain tumors, as present therapeutic options are limited (Jain 2018). For most locally advanced solid tumors, radiation treatment is crucial component of routine treatment, and chemotherapeutic drugs are usually given at the same time for better local tumor control (Kong, et al. 2017).

Hypoxia is a prevalent hallmark of many malignant neoplasms, and it has been identified as a main cause of malignant tissue’s radioresistance. The direct assessment of intratumoral oxygen tension and diagnostic radio imaging techniques, such as position emission tomography (PET) and single-photon-emission computed tomography, have all been reported as ways to demonstrate intratumoral hypoxia (Hompland et al. 2021; Lee et al. 2014). Solid tumors are composed of areas with a high number of hypoxic cells compared to surrounding normal tissue, with significant heterogeneity within the tumor (Muz et al. 2015).

Increased glucose transport, angiogenesis, and anaerobic glycolysis are all hypoxia responses. Hypoxia-inducible factor-1 (HIF-1), especially HIF-1α binds to hypoxia response elements (HREs) and coordinates with many of these responses. HREs are found in the promoter or enhancer regions of target genes such as the angiogenic factor vascular endothelial growth factor (VEGF), erythropoietin (EPO), glucose transporter-1 (GLUT1), and several glycolytic enzymes (Abou Khouzam et al. 2021). VEGF-A is the most potent hypoxia pro-angiogenic factor that binds to VEGFR-1 and VEGFR-2 on endothelial cells and promotes its growth and survival (Schito 2019).

Caveolin-1 (Cav1) has been linked to cellular transport, signal transduction, and human malignancies. Cav1 expression has been reported to be abnormally changed in a wide range of malignancies, with growing evidence pointing to Cav1 overexpression, especially at higher tumor stages. In a number of malignancies, Cav1 overexpression is connected to aggressive clinical behavior and bad prognosis (Mao et al. 2016). Cav1’s role in hypoxia, on the other hand, has yet to be determined. Cav1 has also been demonstrated to enhance survival of tumor cells after RT. Cav1 behaved as a pro-survival factor in pancreatic and lymphoblastoid cancer cells, mediating resistance to damaging consequences of RT (Ketteler and Klein 2018).

The majority of clinical efforts to combat tumor hypoxia have provided conflicting results. Because of better knowledge of the biological mechanisms driven by hypoxia and the disclosure of new hypoxia biomarkers, targeting hypoxia has become more plausible (Zhang et al. 2016). The main target of this study was to assess the circulating serum hypoxia biomarkers HIF-1α, VEGF, osteopontin, erythropoietin, caveolin-1, GLUT-1, and LDH pre- and post-radiotherapy in patients with brain tumors.

## Subjects and Methods

The present study included 120 subjects. They were divided into two groups: group I: 40 healthy subjects as a control group matched in age and sex with patient’s group, group II: 80 brain tumor patients (BTPs) were subdivided into glioblastoma subgroup: it included 40 glioblastoma patients. Meningioma subgroup: It included 40 malignant meningioma patients.

Brian tumor patients were chosen from those admitted to Departments of Neurosurgery & Clinical Oncology, Faculty of Medicine, and Department of Cancer Management and Research, Medical research Institute, Alexandria University, Alexandria, Egypt, in the period between April 2018 and September 2019. This study was conducted following the Declaration of Helsinki and approved by the Ethical Committee of the Medical Research Institute, Alexandria University, Alexandria, Egypt. An informed written consent was obtained from every subject prior to his participation in the study.

Glioblastoma patients were treated with radiotherapy dose 59.4–60 Gy in conventional fractionation (1.8–2 Gy per fraction). Malignant meningioma patients were treated with radiotherapy dose of ≥ 50 Gy in 1.8–2 Gy fractions.

### Inclusion Criteria

Patients were more than 18 years old, all primary brain tumor patients were subjected to surgery. Patients did not receive radiotherapy to the brain tumor or to any tumor elsewhere.

### Exclusion Criteria

Subjects with autoimmune diseases, diabetes, heart diseases, asthma, sepsis, hemolytic disorder, intravascular coagulation syndrome, pulmonary thromboembolism, chronic renal failure, acute hepatic failure, and smokers were excluded from the study.

### Blood Samples Collection

A total of two venous blood samples were collected from every patient in patients’ group. One blood sample was collected before radiotherapy, and the other was collected after the end of radiotherapy. One sample was collected from healthy controls.

The blood sample (5 ml) was allowed to coagulate for 10–20 min at room temperature. It was centrifuged for 20 min at 2000–3000 RPM. The supernatant was collected carefully. Serum was stored at − 80 °C until used. Serum biomarkers (HIF-1α, (ng/ml) VEGF (pg/ml), osteopontin (ng/ml), erythropoietin (pg/ml), caveolin-1(ng/ml), GLUT-1 (ng/ml), and LDH (U/L)) were assayed by enzyme linked immunosorbent assay (ELISA) according to manufactures protocol (Cloud Clone Corp., USA).

### Statistical Analyses

The IBM SPSS software program version 20 (IBM Corporation, Armonk, NY, USA) was used to analyze the data. Qualitative data were described using number and percent. Quantitative data were described using range (minimum and maximum), mean, and standard deviation. The independent *t* test was used to compare two independent populations, and the paired *t* test was used to compare two dependent populations. The significance of the acquired results was assessed at 5% level.

## Results

### Demographic Data of Brain Tumor Patients (BTPs) and Controls

Range and mean ± S.D. of age of BTPs and healthy subjects were presented in Table 1. The difference in mean age and % of sex distribution between BTPs and control group were statistically non-significant (*P* = 0.399 and *P* = 0.715 respectively) (Table 1).

**Table 1:** Demographic data of brain tumor patients (BTP) and controls

	**Brain tumor patients** **(*n* = 80)**	**Control group** **(*n* = 40)**	***p***
**Age (years)**			
Range	42.0–59.0	43.0–56.0	0.399
Mean ± SD	49.49 ± 4.23	48.57 ± 4.07	
**Sex: *N* (%)**			
Male	58 (72.5%)	28 (70%)	0.715
Female	22 (27.5%)	12 (30%)	
**Tumor size (cm)** **Glioblastoma**		–	
≤ 5	35 (87.5%)		
> 5	5 (12.5%)		
**Meningioma**		–	
≤ 5	40 (100%)		
> 5	0 (0%)		

*P p* value comparing between BTPs and controls

#### Tumor Size

In glioblastoma patients, it was ≤ 5 cm in 87.5% of patients, while it was > 5 cm in 12.5% of patients. In malignant meningioma patients, tumor size in all patients was ≤ 5 cm (Table 1).

### Hypoxia Biomarkers in Brain Tumors Patients(BTPs)

Hypoxia biomarkers were measured by ELISA in serum of BTPs and control group. The results were showed in Table 2.

**Table 2:** Comparison between the studied biomarkers in brain tumor patients and controls

**HIF-1α (ng/ml)**	**Control group (*n* = 40)**	**Glioblastoma subgroup (*n* = 40)**		**Meningioma subgroup (*n* = 40)**	
		Before radiotherapy	After radiotherapy	Before radiotherapy	After radiotherapy
Range	0.78–1.89	1.57–11.27	1.90–15.61	1.39–3.92	0.97–2.83
Mean ± SD	1.41 ± 0.32	2.61 ± 1.94	4.34 ± 3.30	2.30 ± 0.67	1.87 ± 0.58
P1		< 0.001^*^	< 0.001^*^	< 0.001*	0.004*
P2		< 0.001^*^		< 0.001^*^	
P3				< 0.001^*^	
**VEGF (pg/ml)**					
Range	383.8–993.6	1096.5–4149.2	1727.2–5121.0	1165.9–2976.4	909.2–1989.3
Mean ± SD	790.2 ± 147.4	1790.0 ± 591.7	2529.4 ± 736.5	1519.7 ± 526.6	1341.6 ± 299.9
P1		< 0.001*	< 0.001*	< 0.001*	< 0.001*
P2		< 0.001*		< 0.001*	
P3				< 0.001*	
**OPN (ng/ml)**					
Range	13.65–32.93	23.29–68.31	42.20–80.31	24.15–45.84	37.07–65.23
Mean ± SD	21.60 ± 5.33	37.14 ± 8.57	64.90 ± 10.23	35.58 ± 5.90	50.73 ± 8.24
P1		< 0.001*	< 0.001*	< 0.001*	< 0.001*
P2		< 0.001*		< 0.001*	
P3				0.014*	
**EPO (pg/ml)**					
Range	83.31–126.1	167.4–317.5	124.2–190.0	145.8–305.6	120.3–210.0
Mean ± SD	108.1 ± 10.04	241.9 ± 46.95	156.7 ± 19.56	223.3 ± 40.14	160.3 ± 22.51
P1		< 0.001*	< 0.001*	< 0.001*	< 0.001*
P2		< 0.001*		< 0.001*	
P3				0.586	
**Cav1 (ng/ml)**					
Range	5.49–8.88	5.60–13.02	3.85–15.0	6.02–13.69	6.11–11.93
Mean ± SD	7.29 ± 0.93	8.07 ± 1.69	9.16 ± 2.45	8.11 ± 1.74	8.56 ± 1.56
P1		0.092	0.005*	0.094	0.007 *
P2		0.011*		0.421	
P3				0.557	
**GLUT-1 (ng/ml)**					
Range	5.92–8.95	7.23–15.66	6.72–17.88	7.05–11.14	8.31 – 13.22
Mean ± SD	7.63 ± 0.73	9.0 ± 1.64	10.65 ± 2.0	8.78 ± 1.27	10.58 ± 1.38
P1		0.002*	< 0.001*	0.003*	< 0.001*
P2		< 0.001*		< 0.001*	
P3				0.713	
**LDH (U/L)**					
Range	200.0–890.0	815.0–2345.0	1205.0–3825.0	745.0–3425.0	255.0–1915.0
Mean ± SD	578.57 ± 279.62	1498.18 ± 437.20	1908.64 ± 729.85	1544.58 ± 757.87	1185.0 ± 541.50
P1		< 0.001*	< 0.001*	0.005*	0.014*
P2		0.027*		0.045*	
P3				0.013*	

*P1*: *p* value for comparing between control group with each studied subgroup

*P2*: *p* value for comparing between before and after radiotherapy in the same subgroup

*P3*: *p* value for comparing between glioblastoma and meningioma subgroups after radiotherapy

^*^Statistically significant at *p* ≤ 0.05

### Serum HIF-1α (ng/ml)

In glioblastoma patients pre-RT, the mean ± SD of serum HIF-1α was 2.61 ± 1.94 that was increased to 4.34 ± 3.30 post-RT, while it was 1.41 ± 0.32 in healthy controls. Statistical analysis showed a significant increase in serum HIF-1α in glioblastoma subgroup either prior to or following RT in comparison to healthy volunteers (p1 < 0.001and < 0.001 respectively). In addition, serum HIF-1α was significantly increased after RT (p2 < 0.001).

Regarding meningioma patients, the mean ± SD of serum HIF-1α was 2.30 ± 0.67 before RT that was decreased to 1.8 ± 0.58 after RT. Serum HIF-1α levels were Statistically increased either prior to or following RT in comparison to healthy volunteers (p1 < 0.001 and 0.004 respectively). However, this biomarker was significantly decreased after RT (p2 < 0.001). HIF-1α levels after RT in glioblastoma patients were significantly higher than meningioma patients (p3 < 0.001).

### Serum VEGF (pg/ml)

In glioblastoma patients pre-RT, the mean ± SD of serum VEGF was 1790 ± 591.7 that was increased to 2529.4 ± 736.5 post-RT, while it was 790.2 ± 147.4 in healthy controls. Statistical analysis showed a significant increase in serum VEGF in glioblastoma subgroup either prior to or following RT in comparison to healthy volunteers (p1 < 0.001 and < 0.001 respectively). Moreover, serum VEGF was significantly increased after RT (p2 < 0.001).

Regarding meningioma subgroup, the mean ± SD of serum VEGF was 1519.7 ± 526.6 before RT that decreased to 1341.6 ± 299.9 after RT. Serum VEGF levels was increased either prior to or following RT in comparison to healthy volunteers (p1 < 0.001 and < 0.001 respectively). However, this biomarker was significantly decreased after RT (p2 < 0.001). VEGF after RT in glioblastoma subgroup was significantly higher than meningioma patients (p3 < 0.001).

### Serum Osteopontin (OPN), (ng/ml)

In glioblastoma patients pre-RT, the mean ± SD of serum OPN was 37.14 ± 8.57 that was increased to 64.90 ± 10.23 post-RT. While, it was 21.60 ± 5.33 in healthy controls. Statistical analysis showed a significant increase in serum OPN in glioblastoma subgroup either prior to or following RT in comparison to healthy volunteers (p1 < 0.001 and < 0.001, respectively). Moreover, serum OPN was significantly increased after RT (p2 < 0.001).

Regarding meningioma subgroup, the mean ± SD of serum OPN was 35.58 ± 5.90 before RT that was increased to 50.73 ± 8.24 after RT. Analysis of data revealed a significant increase in serum OPN in meningioma patients either pre- or post-RT when compared to healthy volunteers (p1 < 0.001 and 0.001, respectively). Moreover, serum OPN was significantly increased post-RT when compared to pre-RT (p2 < 0.001). Serum OPN after RT in glioblastoma patients was significantly higher than in meningioma patients (p3 = 0.014).

### Serum Erythropoietin (EPO), (pg/ml)

In glioblastoma patients pre-RT, the mean ± SD of serum EPO was 241.9 ± 46.95 that was decreased to 156.7 ± 19.56 post-RT, while it was 108.1 ± 10.04 in healthy controls. Statistical analysis showed a significant increase in serum EPO in glioblastoma subgroup either prior to or following RT in comparison to healthy volunteers (p1 < 0.001 and < 0.001, respectively). However, serum EPO was significantly decreased after RT (p2 < 0.001).

Regarding meningioma subgroup, the mean ± SD of serum OPN was 223.3 ± 40.14 pre-RT that was decreased to 160.3 ± 22.51 post-RT. Statistical analysis of data revealed a significant increase in serum EPO in meningioma patients either before or after RT when compared to healthy volunteers (p1 < 0.001 and < 0.001, respectively). Moreover, serum EPO was significantly decreased after RT when compared to before RT (p2 < 0.001). Comparison between glioblastoma and meningioma patients showed insignificant difference in serum EPO between the two subgroups after RT (p3 = 0.586).

### Serum Caveolin-1(Cav1), (ng/ml)

In glioblastoma patients pre-RT, mean ± SD of serum Cav1 was 8.07 ± 1.69 that was increased to 9.16 ± 2.45 post-RT, while it was 7.29 ± 0.93 in healthy controls. Serum Cav1 showed a significant increase in glioblastoma subgroup after RT in comparison to healthy volunteers (p1 < 0.001). In addition, serum Cav1 increased significantly after RT (p2 = 0.011).

In meningioma subgroup, the mean ± SD of serum Cav-1 was 8.11 ± 1.74 pre-RT that was increased to 8.56 ± 1.56 post-RT. Serum Cav1 was insignificantly different either pre- or post-RT (p2 = 0.421). Serum Cav1 after RT in glioblastoma patients was insignificantly higher than in meningioma patients (p3 = 0.557).

### Serum GLUT-1(ng/ml)

In glioblastoma patients pre-RT, the mean ± SD of serum GLUT-1 was 9.0 ± 1.64 that was increased to 10.65 ± 2.0 post-RT, while it was 7.63 ± 0.73 in healthy controls. Statistical analysis showed a significant increase in serum GLUT-1 in glioblastoma subgroup either prior to or following RT in comparison to healthy volunteers (p1 = 0.002 and < 0.001 respectively). Serum GLUT-1 was significantly increased after RT (p2 < 0.001).

In meningioma patients pre-RT, the mean ± SD of serum GLUT-1 was 8.78 ± 1.27 that was increased to 10.58 ± 1.38 post-RT. Results showed significant increase in serum GLUT-1 in meningioma patients either prior to or following RT when compared to healthy volunteers (p1 = 0.003, < 0.001 respectively). Moreover, serum GLUT-1 was significantly increased after RT when compared to before RT (p2 < 0.001). After RT, serum GLUT-1 was insignificantly higher in glioblastoma patients in comparison to meningioma patients (p3 = 0.713).

### Serum LDH (U/L)

In glioblastoma patients pre-RT, the mean ± SD of serum LDH was 1498.18 ± 437.20 that was increased to 1908.64 ± 729.85 post-RT, while it was 578.57 ± 279.62 in healthy controls. Statistical analysis showed a significant increase in serum LDH in glioblastoma subgroup either prior to or following RT in comparison to healthy volunteers (p1 < 0.001and < 0.001 respectively). Moreover, serum LDH was significantly increased after RT (p2 = 0.027).

Regarding meningioma patients, mean ± SD of serum LDH was 1544.58 ± 757.87 in meningioma patients pre-RT that was decreased to 1185.0 ± 541.50 post-RT. Serum LDH levels were increased either prior to or following RT in comparison to healthy volunteers (p1 = 0.005 and = 0.014 respectively). However, serum LDH was significantly decreased after RT (p2 = 0.045). Serum LDH after RT in glioblastoma patients was significantly higher than in meningioma patients (p3 = 0.013).

## Discussion

Radiotherapy can be used as a primary treatment or in conjunction with surgical resection in patients with brain tumors. The most prevalent technique is standard fractionated external beam radiotherapy; hypofractionated radiotherapy can enhance survival in patients with brain tumors (Roy and Bandyopadhyay 2018). Hypoxia, a pathophysiologic feature of solid tumors, is a primary cause of irradiation resistance. The hypoxia response is regulated by HIF-1α (Monteiro et al. 2017).

The current study revealed a significant increase in serum HIF-1α in glioblastoma (GB) subgroup either prior to or following RT in comparison to healthy volunteers. Indicating that, this parameter is a marker of malignancy. In addition, this biomarker significantly increased after exposure to RT when compared to before RT. This indicates that GB is in a state of hypoxia. Our findings are consistent with previous studies (Reszec et al. 2013; Lo Dico et al. 2018). Irie et al. (2004) used immunohistochemistry to assess the degree of HIF-1α expression in 60 patients with GB. HIF-1 expression was positive in all tissue samples. They proposed that the level of HIF-1α may be used to predict tumor radioresistance. In the current study, the increase of HIF-1α after exposure to RT in GB patients is explained that GB is a malignant tumor that is highly aggressive and hypoxic. These tumors have an exceptionally bad prognosis, mainly due to therapy resistance and tumor recurrence (Lee et al. 2018). Hypoxia is a primary mechanism of tumor resistance in GB, and radiotherapy only provides palliation (Sheehan et al. 2010).

Radiotherapy kills cancer cells through induction of DNA damage through formation of free radicals. Free radicals can react with oxygen molecules, resulting in a persistent DNA damage. Oxygen fixes DNA damage in this way (Horsman et al. 2009). Hypoxia prevents DNA damage fixation and hence, it is a significant reason of irradiation resistance (Hoogsteen et al. 2007).

The majority of primary cancers of the central nervous system are treated with RT. However, radioresistance severely limits the efficacy of RT. The relative radioresistance of glioblastoma-initiating cells (GICs) has been related to the poor prognostic character of glioblastoma. GIC resistance is thought to have a role in the poor response to radiation and chemotherapy, as well as the inevitable tumor recurrence. Although the specific mechanism of treatment resistance is uncertain, GICs’ intrinsic hyperactivation of the PI3K/Akt and PTEN pathways, as well as enhanced activation of DNA damage checkpoint pathways, are thought to play a role (Frosina 2021).

In the present study, there was a significant increase in serum VEGF either before or after RT in both subgroups. Also, there was a significant increase after exposure to RT in glioblastoma subgroup on comparing to meningioma subgroup suggesting that VEGF expression is strongly induced by hypoxia within GB. In a series of 40 glioblastoma patients, Reynés et al. (2011) found that the serum levels of VEGF were twofold higher in patients compared with healthy controls. Previous studies (Oehring et al. 1999; Krcek et al. 2017) stated that the increase of VEGF after RT indicates radioresistance and treatment failure. GB, considered the most vascularized brain tumor and VEGF, has a major significance in angiogenesis (Hottinger et al. 2014). To force neoangiogenesis and development, GB produces a lot of VEGF. Hypoxia inducible factors, which are activated by inadequate blood supply, are some stimuli for the release of VEGF (Liao and Johnson 2007). By increasing angiogenesis and/or vasculogenesis, HIF-1 is responsible for vascular protection, tumor blood and nutrition supply recovery, and tumor recurrence after irradiation. Preclinical evidence strongly implies that tumor resistance to irradiation in GB patients is due to HIF-1–mediated vascular protection (Meijer et al. 2012). VEGF levels beyond a certain threshold appear to be a survival factor for irradiated cancer cells, particularly GB cells (Knizetova et al. 2008).

In the current study, both HIF-1α and VEGF were significantly decreased after RT when compared to before RT in meningioma patients. These results were in agreement with previous studies (Kaynar et al. 2008; Jensen and Lee 2012). The growth of meningiomas and the development of their vascularization are strongly related to the activity of various biochemical factors. According to some reports, there is no link between VEGF expression levels and tumor size, location, or histological subtype, and there is no link between VEGF levels and tumor vascularization (Barresi 2011; Dharmalingam et al. 2013). Moreover, there was no difference in the degree of VEGF expression between non-malignant and malignant meningiomas. Gliomas’ infiltrative nature and meningiomas’ expansive distorted growth result in a different type of tumor-feeding vessel formation. Meningiomas require the construction of new vessels to enter within the circumscribed tumor, whereas gliomas perform the existing blood vessels in the brain (Śniegocki et al. 2021).

Moreover, patients with meningioma have high life expectancy. These tumors have a generally good prognosis, with local control rates as high as 90% after 10 years (Bender et al. 2020). After particle and photon irradiation, Mozes et al. (2017) discovered a significant tumor volume decrease after 1 and 2 years of follow-up with conventional external beam radiation. It appears to be an effective and safe primary or adjuvant treatment for meningiomas.

Comparison between meningioma and glioblastoma subgroups according to serum OPN showed that this parameter was significantly increased after exposure to radiotherapy in both subgroups with significant elevation in glioblastoma than in meningioma patients. Association of significant elevation of serum osteopontin (OPN) with aggressive and hypoxic glioblastoma subgroup supports the function of OPN in cancer aggressiveness and high serum OPN levels may be a marker of hypoxia. High serum OPN level is found as a poor prognostic indicator in GBMs (Sreekanthreddy et al. 2010). Henry et al. revealed that OPN plays a role in the initiation of DNA repair in response to irradiation in GBM cells and hence induce GBM radioresistance (Henry et al. 2016).

Le et al. (2003) identified osteopontin as a potential hypoxia marker. OPN levels in the serum have been linked to bad outcome in glioblastoma patients, according to a previous study (Schuhmann et al. 2010). Glioma cell migration and invasion are induced by OPN (Yan et al. 2010; Lu et al. 2012). OPN had elevated expression levels in the cerebrospinal fluid (CSF) of patients with GB, according to a recent study by Kohata et al. (2020).

The present study revealed a significant increase in serum erythropoietin (EPO) in BTPs either prior to or following RT in comparison to control group. Higher serum EOP levels in BTPs indicate tumor-related anemia, which reduces the blood’s oxygen-carrying capacity and causes tissue hypoxia. Hypoxia is a known hallmark of radioresistance; thus, researchers predict that elevated endogenous erythropoietin levels may be used as a hypoxia marker, causing the tumor to become more aggressive and perhaps impairing treatment effects (Lazzari and Silvano 2020).

Erythropoietin (EPO) is a hormone that controls the human body’s daily production of 200 billion new RBCs. EPO enhances the survival, proliferation, and differentiation of erythroid progenitor cells into mature erythrocytes. EPO is produced in the adult kidney’s interstitial cells and is induced by hypoxia Suresh et al. (2020), Tsifsoglou (2021). In the current study, EPO levels declined significantly after RT, but they remained significantly higher than normal control values, demonstrating that patients can still respond to hypoxia by increasing serum EPO levels. Cytotoxic treatment which causes damage to EPO-producing cells, is one possible explanation for EPO decrease after RT (Schapira et al. 1990).

In the present study, serum Cav1 was increased significantly after RT when compared to before RT in GB patients and was higher than its levels in meningioma patients. Association of higher Cav1 levels with hypoxic tumor GB indicates its role in tumor hypoxia. Castillo Bennett et al. (2018) evaluated whether hypoxia induced expression of Cav1 in metastatic cancer cells. He and his colleagues found that hypoxia increases Cav1 protein levels in a HIF-1α dependent manner. On the other hand, Chen et al. (2019) examined HIF-1α and Cav1 expression in GB. In 2019, they observed that the expression of Cav1 was significantly correlated with high HIF-1α expression. In 2021, Chen et al. (2021) identified Cav1 as an important regulator of glioma cell proliferation contributing to glioma development and progression.

In the current study, serum GLUT-1 significantly increased after RT when compared to before treatment in both subgroups. Mamun et al. (2020) discovered that during hypoxia, glucose absorption increased by 19%, which was linked to an increase in GLUT-1 protein production. Additionally, GLUT1 translocation was linked to a drop in intracellular ATP, but not to an increase in HIF-1. Furthermore, ATP-stimulated translocation of GLUT-1 to the plasma membrane could be a part of a feedback process between metabolic status and glucose uptake, which may enhance glucose uptake in hypoxia. According to Zhao et al. (2016) radiotherapy stimulates glucose metabolism and GLUT-1 expression, and overexpression of GLUT-1 makes breast cancer cells resistant to radiation.

In the current study, LDH revealed a significant increase before and after RT in glioblastoma patients when compared to healthy controls and was increased after RT when compared to before RT. Suggesting that LDH may be a potential marker of tumor-associated hypoxia in GB. Xie et al. (2019) explored that LDH mRNA expression is increased in hypoxic condition and LDH expression is regulated transcriptionally by HIF-1. LDH is a tetrameric enzyme that accelerates the rate of pyruvate to lactate interconversion. Microenvironment of the tumor is frequently hypoxic and acidic as a result of uncontrolled tumor growth. Hypoxia causes HIF-1 to enhance LDH transcription, and LDH overexpression causes lactate synthesis, lowering pH levels. It has also been discovered that an acidic pH enhances angiogenesis and migration of glioma stem cells via inducing glioma stem cell markers (Valvona et al. 2016). Serum LDH was significantly decreased in meningioma patients after RT. Although the exact mechanism of lowering serum LDH in meningioma is unknown, further research is required if LDH levels could have some prognostic value in following up tumor recurrence.

Limitation of the present study includes lack of radiological response to radiotherapy and survival analysis that may interfere with interpretation of the results of this study.

## Conclusions

Association of significant elevation of hypoxia biomarkers either pre- or post-RT with aggressive tumor such as glioblastoma indicates that they are markers of malignancy and may have a role in tumor development and progression.

## Recommendations

Circulating levels of hypoxia biomarkers should be one of the indicators in the postoperative radiation regimen for brain tumors patients. To improve the efficacy of radiotherapy in BTPs, therapeutic approaches to overcome tumor hypoxia are required. To verify our findings, a bigger clinical study should be conducted including survival analysis.

## Data Availability

The datasets generated during and/or analyzed during the current study are available from the corresponding author on reasonable request.

## Abbreviations

RT: Radiotherapy
BTP: Brain tumor patient
HIF-1α: Hypoxia inducible factor-1 α
VEGF: Vascular endothelial growth factor
OPN: Osteopontin
EPO: Erythropoietin
Cav1: Caveolin-1
GLUT-1: Glucose transporter-1
GB: Glioblastoma
